# Genome-wide analysis of the MADS-box gene family involved in salt and waterlogging tolerance in barley (*Hordeum vulgare* L.)

**DOI:** 10.3389/fpls.2023.1178065

**Published:** 2023-05-09

**Authors:** Feifei Wang, Zhenxiang Zhou, Liang Zhu, Yangyang Gu, Baojian Guo, Chao Lv, Juan Zhu, Rugen Xu

**Affiliations:** Key Laboratory of Plant Functional Genomics of the Ministry of Education, Jiangsu Key Laboratory of Crop Genomics and Molecular Breeding, Jiangsu Co-Innovation Center for Modern Production Technology of Grain Crops, Institutes of Agricultural Science, Yangzhou University, Yangzhou, China

**Keywords:** MADS-box, barley, salt stress, waterlogging stress, protein-protein interaction

## Abstract

MADS-box transcription factors are crucial members of regulatory networks underlying multiple developmental pathways and abiotic stress regulatory networks in plants. Studies on stress resistance-related functions of MADS-box genes are very limited in barley. To gain insight into this gene family and elucidate their roles in salt and waterlogging stress resistance, we performed genome-wide identification, characterization and expression analysis of MADS-box genes in barley. A whole-genome survey of barley revealed 83 MADS-box genes, which were categorized into type I (Mα, Mβ and Mγ) and type II (AP1, SEP1, AGL12, STK, AGL16, SVP and MIKC*) lineages based on phylogeny, protein motif structure. Twenty conserved motifs were determined and each *HvMADS* contained one to six motifs. We also found tandem repeat duplication was the driven force for *HvMADS* gene family expansion. Additionally, the co-expression regulatory network of 10 and 14 *HvMADS* genes was predicted in response to salt and waterlogging stress, and we proposed *HvMADS11*,13 and 35 as candidate genes for further exploration of the functions in abiotic stress. The extensive annotations and transcriptome profiling reported in this study ultimately provides the basis for MADS functional characterization in genetic engineering of barley and other gramineous crops.

## Introduction

About 12,000 years ago in the Near East, humans transitioned from hunter-gathering to agriculture-based societies. Barley (*Hordeum vulgare* L.) was a founder crop in this process and was also one of the earliest domesticated crops (Diamond, 2002; Zohary et al., 2012). Barley (2n = 14) is a diploid member of the grass family, making it a natural model for the genetics and genomics of the Triticeae tribe, including polyploid wheat and rye. With a haploid genome size of ~5.3 Gb in seven chromosomes, barley is one of the largest diploid genomes sequenced to date, making it challenging to work with from a genetics, genomics, and breeding perspective (Mascher et al., 2017; Jayakodi et al., 2020). However, recent advances in sequencing technology have led to high-quality genome assembly and annotation by the Leibniz Institute of Plant Genetics and Crop Plant Research in 2021 (Hordeum vulgare Morex V3, 2021; Mascher et al., 2021). Further, large-scale RNA-seq analyses provided insights into the expression patterns of homoologous genes at different developmental stages and under a variety of stress conditions, building a rich resource for more detailed analyses.

Transcription factors bear the potential for trait fine-tuning and crop improvement in response to biotic or abiotic stress (Martinez-Ainsworth and Tenaillon, 2016; Schilling et al., 2020). MADS-box genes constitute one of the largest families of plant transcription factors (Riechmann et al., 2000). MADS is an acronym derived from the four founding members of the family: MCM1 from *Saccharomyces cerevisiae* (baker’s yeast), AGAMOUS (AG) from *Arabidopsis thaliana*, DEFICIENS (DEF) from *Antirrhinum majus* (snapdragon), and SRF from *Homo sapiens* (Schwarz-Sommer et al., 1990; Shore and Sharrocks, 1995; Lawton-Rauh et al., 2000; Schilling et al., 2018). Throughout the eukaryotes, two types of MADS-box genes are distinguished by the highly conserved, 56–60 amino acid-long, DNA-binding MADS domain (Schwarz-Sommer et al., 1990; Alvarez-Buylla et al., 2000; Gramzow et al., 2010). The type I lineage includes the ARG80/SRF-like domain (Becker and Theissen, 2003). The type II lineage, also termed MIKC-type, comprises MIKC^C^ and MIKC* genes, referring to the typical domain structure of the encoded proteins, including a MADS box domain (M), an intervening domain (I), a keratin-like K-box (K), and a C-terminal domain (C) (Theissen et al., 1996; Kaufmann et al., 2005). The highly conserved M domain has sequence-specific DNA binding activity, which also functions in dimerization and nuclear localization (Immink et al., 2002). The weakly conserved I domain is a regulatory determinant for the formation of DNA-binding dimers (Theissen et al., 2016). The K-box domain, the second most conserved domain after the MADS domain, is defined by conserved regular spacing of hydrophobic residues and can form amphipatic helices involved in protein dimerization, which mediates protein–protein interactions (Yang and Jack, 2004; Callens et al., 2018). The most variable domain is located at the C-terminal end, which is involved in transcriptional activation and the formation of multimeric transcription factor complexes (Honma and Goto, 2001; Becker and Theissen, 2003).

MADS-box genes are involved in virtually all aspects of plant development, including flowering time control, inflorescence architecture, floral organ identity determination, and seed development (Schilling et al., 2020). They have also been reported to function in different abiotic stress responses (Arora et al., 2007; Jia et al., 2018; Castelan-Munoz et al., 2019; Zhao et al., 2021). In tomato, the expression of *SlMBP11* (an AGL15 ortholog) is induced by salt and drought stress. Knocking down this gene makes the lines more sensitive to salt stress conditions than wild-type plants (Guo et al., 2016). Over-expressing *AGL21* in Arabidopsis affects germination rate and makes seeds hypersensitive to salt stress, which suggests AGL21 as a negative regulator of seed germination under salt stress conditions (Yu et al., 2017). AGL16 is found to be a negative regulator of the stress response in Arabidopsis. Loss-of-AGL16 confers resistance to salt stress in seed germination and root elongation, while elevating AGL16 expression confers the opposite phenotypes compared with wildtype (Zhao et al., 2021). Meanwhile, AGL16 directly binds to the CArG motifs in the promoter of *HKT1;1*, *HsfA6a*, and *MYB102* and expresses their expressions (Zhao et al., 2021). CaMADS-downregulated seedlings are more seriously injured than wild-type seedlings after cold, NaCl, and mannitol treatments, which suggests that CaMADS functions as a positive stress-responsive transcription factor in the cold, salt, and osmotic stress signaling pathways (Chen et al., 2019). In *OsMADS25* overexpression lines, the free proline contents are higher, the accumulation of MDA (malonaldehyde) is lower, and genes related to salt stress are significantly upregulated, which clearly demonstrates that OsMADS25 improves salt tolerance by reducing oxidative damages (Wu et al., 2020). Reports about the MADS under waterlogging stress are rare. The expression patterns of the MADS-box gene in *Rhododendron hainanense* under different waterlogging stress conditions were analyzed, and the expressions of *RhMADS22*, *RhMADS24*, *RhMADS25*, *RhMADS27*, *RhMADS33*, *RhMADS39*, *and RhMADS44* were upregulated during different waterlogging treatments, while *RhMADS29 and RhMADS44* were downregulated (Huo et al., 2021).

Investigations on the MADS gene functions in barley are limited. The roles of the grass-specific MADS box gene *ODDSOC2* (*OS2*) in vernalization responses are investigated in barley. Overexpression of *HvOS2* delays flowering and reduces spike, stem, and leaf length in plants. *HvOS2* is repressed by vernalization; meanwhile, the active alleles of the *VRN1* gene (*HvVRN1*) also downregulate *HvOS2* during development (Greenup et al., 2010). The functions of short vegetative phase (SVP)-like MADS-box genes in barley indicate a role in determining meristem identity (Trevaskis et al., 2007). Three SVP-like genes, including *Barley MADS1* (*BM1*), *BM10*, and *Vegetative to Reproductive Transition Gene 2*, are induced by cold but repressed during floral development, suggesting that SVP-like genes suppress floral meristem identity in winter cereals (Trevaskis et al., 2007).

MADS-box genes have been phylogenetically and functionally characterized in a variety of model systems, such as *A. thaliana*, encoding 107 MADS-box genes; *Brachypodium distachyon*, encoding 75 MADS-box genes; *Brassica rapa*, encoding 160 MADS-box genes; and *Oryza sativa*, encoding 75 MADS-box genes (Parenicova et al., 2003; Arora et al., 2007; Wei et al., 2014). To better understand the dynamics of MADS-box gene evolution in barley and to facilitate future research on this important transcription factor family, we provide genome-wide analysis and characterization of MADS-box genes in barley based on the recently released genome of *Hordeum vulgare* Morex V3, 2021. In the present study, whole MADS-box family members from the barley genome were firstly identified and divided into different classes, and the conserved motifs and phylogenetic relationships between these members were systematically analyzed. Additionally, chromosome locations, gene duplication, and syntenic relationship analysis were also investigated. The expression patterns of MADS-box genes and protein interaction networks under salt and waterlogging stress in barley were analyzed. These results contribute to the functional analysis of MADS-box genes and facilitate dissecting the MADS-box gene-mediated molecular mechanisms underlying abiotic stress in barley.

## Materials and methods

### Identication of *HvMADS* genes in barley

To identify the candidate *HvMADS* genes, we downloaded MADS protein domains PF00319 and PF01486 from the pfam (https://pfam.xfam.org/) website to construct a hidden Markov model (HMM) and used this model to search the protein database in the barley genome (*Hordeum vulgare* Morex V3, 2021) by using the HMMER website (https://www.ebi.ac.uk/Tools/hmmer/search/hmmsearch). Then, we used the pfam tool with an e-value of <0.05 and the Conserved Domain Database (CDD) to analyze the left sequence, and those without the PF00319 and PF01486 domains were discarded. All CDSs were translated into amino acid sequences and aligned with all MADS-domain protein sequences of rice and Arabidopsis with Jalview software. Ultimately, 83 *HvMADS* genes were identified. Furthermore, the ExPASy-ProtParam tool (https://web.expasy.org/protparam/) was used to calculate amino acid numbers, molecular weights (MW), and isoelectric point (pI), and instability index.

### Phylogenetic analysis of HvMADSs

Multiple sequence alignments of 83 HvMADS proteins with MADS genes from rice and Arabidopsis were conducted using ClustalW. A neighbor-joining phylogenetic tree was constructed based on the full-length protein sequences using MEGAX software with a bootstrap of 1,000 replications. The phylogenetic tree was further beautified with ChiPlot (https://www.chiplot.online/).

### Gene cluster and protein motif analysis

MEGAX and Jalview were used to compare the sequences of MADS gene family members in barley. The Multiple Expectation Maximization for Motif Elicitation (MEME) online program (https://meme-suite.org/meme/tools/meme) was performed to identify conserved motifs of HvMADS proteins. The conserved motif of HvMADS was displayed by the Gene Structure View in TB tools.

### Chromosomal location and gene duplication

*HvMADS* genes localization on chromosome was visualized by TBtools (Chen et al., 2020). Syntenic relationship of the orthologous MADS genes between *H. vulgare*, *A. thaliana*, *O. sativa*, *Zea mays*, and *Triticum aestivum* were analyzed by the MCScanX software. Gene duplication was also analyzed and displayed by MCScanX in TBtools.

### Cis-acting element analysis of promoter of MADS gene family in barley

The upstream 1.5 kb genomic DNA sequences of each gene were extracted from the barley genome and then submitted to the PlantCARE website (https://bioinformatics.psb.ugent.be/webtools/plantcare/html/) to detect putative cis-regulatory elements.

### Plant treatment and gene expression analysis

NasoNijo (a salt and waterlogging sensitive variety, NN) and TX9425 (a salt and waterlogging tolerant variety, TX) were grown in the same pot (20 cm ∗ 30 cm) in a greenhouse with a day-night temperature of 22 ± 3°C 16 h/8 h day/night regime. Salt and waterlogging stress experiments were carried out separately. The seedlings were grown to the three-leaf stage and treated with 300 mM/L NaCl for 1 h, 24 h, and 10 d or submerged in tap water with 1 cm of water above the soil for 1 h, 72 h, and 2 w. Leaves and roots were sampled for RNA isolation and RNA sequencing. The average expression level of three biological replicates was calculated, and the *HvMDAS* gene expression values were represented by log_2_(fragments per kilobase of exon model per million mapped fragments) and their heatmap was conducted by TBtools. Furthermore, genes with |log_2_(fold change)|>1 and p-value <0.5 were regarded as differentially expressed genes.

### Protein–protein interaction network for HvMADS under salt and waterlogging stress

Based on the transcriptome analysis after salt and waterlogging stress, the differentially expressed genes were chosen as subjects for the protein-protein interaction network. The rice homologous genes corresponding to barley were found by comparison, and the MADS-box protein interaction network of rice was analyzed using the online website String (https://string-db.org/) and visualized by the software Cytoscape (http://www.cytoscape.org/).

## Results

### The barley genome contains 83 MADS-box genes

In total, 89 MADS-box family members were identified in barley using the hidden Markov model, which was built on the MADS-box and K-box domains separately and used to search the recently released barley genome (*Hordeum vulgare* Morex V3, 2021). Two and four genes were deleted to keep only one splice variant from each genomic locus for comparison with online sites, including NCBI-CDD. The remaining candidate genes were analyzed through multiple sequence alignment and phylogenetic relationship analysis, which differentiated type I and type II MADS-box genes. Ultimately, 83 MADS genes were identified, with 46 of them belonging to type I and 37 belonging to type II based on the MADS-box and K-box domains. Gene names were determined according to their position on the chromosome. The detailed information on genes and proteins is listed in **Table 1**. The amino acid length of 54% of the 83 HvMADS proteins ranged from 200 to 300 bp, and 24% had between 300 and 400 bp, with HvMADS34 having the shortest protein length (73 amino acids) and HvMADS75 having the longest length (443 amino acids). According to amino acid length, we predicted the molecular weight of all members, which ranged from 8.3 to 46.9 kDa. Meanwhile, the isoelectric point was within the range of 4.4 to 10.8.

**Table 1:** Detail information of HvMADSs.

Gene name	Gene ID	Chr	Location	Protein length (aa)	Molecular Weight (KDa)	Iso-electric Point	Instability Index	Pfam	Type
*HvMADS1*	HORVU.MOREX.r3.1HG0002750.1	1H	5520201–5520752	183	19.85	5.55	58.71	PF00319	I
*HvMADS2*	HORVU.MOREX.r3.1HG0008600.1	1H	20382140–20387164	237	26.88	6.81	55.89	PF00319; PF01486	II
*HvMADS3*	HORVU.MOREX.r3.1HG0008610.1	1H	20454437–20460812	234	26.83	8.46	53.1	PF00319; PF01486	II
*HvMADS4*	HORVU.MOREX.r3.1HG0024860.1	1H	99872268–99878935	252	28.14	9.28	52.02	PF00319; PF01486	II
*HvMADS5*	HORVU.MOREX.r3.1HG0031260.1	1H	153900663–153908287	266	30.35	9.34	48.88	PF00319; PF01486	II
*HvMADS6*	HORVU.MOREX.r3.1HG0054220.1	1H	362447175–362456082	115	13.03	9.55	44.75	PF00319	I
*HvMADS7*	HORVU.MOREX.r3.1HG0065060.1	1H	427165183–427169503	209	24.42	9.08	65.61	PF00319; PF01486	II
*HvMADS8*	HORVU.MOREX.r3.1HG0065500.1	1H	429027618–429031539	252	27.81	9.43	55.56	PF00319; PF01486	II
*HvMADS9*	HORVU.MOREX.r3.2HG0119930.1	2H	69290056–69291096	346	38.64	8.37	55.81	PF00319	I
*HvMADS10*	HORVU.MOREX.r3.2HG0119950.1	2H	69515817–69516371	184	20.57	9.56	51.53	PF00319	I
*HvMADS11*	HORVU.MOREX.r3.2HG0127410.1	2H	111586811–111611184	289	32.67	6.68	61.24	PF00319; PF01486	II
*HvMADS12*	HORVU.MOREX.r3.2HG0156870.1	2H	381298545–381307819	276	31.84	8.97	66.83	PF00319; PF01486	II
*HvMADS13*	HORVU.MOREX.r3.2HG0170570.1	2H	491665665–491667698	202	23.45	6.98	55.44	PF00319; PF01486	II
*HvMADS14*	HORVU.MOREX.r3.2HG0173440.1	2H	512332014–512347171	240	27.53	8.46	46.38	PF00319; PF01486	II
*HvMADS15*	HORVU.MOREX.r3.2HG0190700.1	2H	600377771–600379968	276	31.95	8.4	65.29	PF00319; PF01486	II
*HvMADS16*	HORVU.MOREX.r3.2HG0195730.1	2H	614114340–614115413	357	38.83	6.32	44.83	PF00319	I
*HvMADS17*	HORVU.MOREX.r3.2HG0206640.1	2H	639677225–639678474	391	43.71	5.39	59.73	PF00319	I
*HvMADS18*	HORVU.MOREX.r3.2HG0206660.1	2H	639773343–639774235	241	27.37	9.62	50.14	PF00319	I
*HvMADS19*	HORVU.MOREX.r3.3HG0243300.1	3H	88737672–88738241	189	21.33	8.84	50.85	PF00319	I
*HvMADS20*	HORVU.MOREX.r3.3HG0243770.1	3H	93103462–93111596	271	30.81	8.99	62.75	PF00319; PF01486	II
*HvMADS21*	HORVU.MOREX.r3.3HG0244110.1	3H	96016168–96016923	251	27.67	8.93	50	PF00319	I
*HvMADS22*	HORVU.MOREX.r3.3HG0286170.1	3H	469468470–469470793	196	22.27	8.88	42.03	PF00319; PF01486	II
*HvMADS23*	HORVU.MOREX.r3.3HG0302630.1	3H	549825183–549826190	335	36.36	6.34	50.82	PF00319	I
*HvMADS24*	HORVU.MOREX.r3.3HG0307160.1	3H	564126570–564128730	209	24.08	7.13	45.79	PF00319; PF01486	II
*HvMADS25*	HORVU.MOREX.r3.3HG0310820.1	3H	575618577–575618819	80	9.06	10.84	81.61	PF00319	I
*HvMADS26*	HORVU.MOREX.r3.3HG0311160.1	3H	576849880–576879501	172	19.02	9.48	50.56	PF00319	I
*HvMADS27*	HORVU.MOREX.r3.3HG0313860.1	3H	584089896–584090888	330	36.38	5.35	54.63	PF00319	I
*HvMADS28*	HORVU.MOREX.r3.3HG0330170.1	3H	618175709–618176833	374	40.56	4.71	54.14	PF00319	I
*HvMADS29*	HORVU.MOREX.r3.3HG0330180.1	3H	618190146–618191072	308	33.32	9.24	47.04	PF00319	I
*HvMADS30*	HORVU.MOREX.r3.3HG0330190.1	3H	618254032–618254787	251	26.56	8.62	62.02	PF00319	I
*HvMADS31*	HORVU.MOREX.r3.4HG0334170.1	4H	7835707–7837714	350	38.11	4.87	54.75	PF00319	I
*HvMADS32*	HORVU.MOREX.r3.4HG0362740.1	4H	207304363–207307893	387	43.02	5.96	52.53	PF00319	I
*HvMADS33*	HORVU.MOREX.r3.4HG0396400.1	4H	532470424–532478567	250	28.67	7.7	65.51	PF00319; PF01486	II
*HvMADS34*	HORVU.MOREX.r3.4HG0396410.1	4H	532600690–532600911	73	8.30	10.43	47.98	PF00319	I
*HvMADS35*	HORVU.MOREX.r3.4HG0406150.1	4H	573194639–573211101	227	25.61	6.23	55.05	PF00319; PF01486	II
*HvMADS36*	HORVU.MOREX.r3.4HG0412460.1	4H	594538879–594559903	212	24.11	8.27	56.44	PF00319; PF01486	II
*HvMADS37*	HORVU.MOREX.r3.4HG0413180.1	4H	596284994–596302716	168	18.36	6.61	53.58	PF00319	I
*HvMADS38*	HORVU.MOREX.r3.5HG0419840.1	5H	1294611–1302389	232	26.37	9.05	52.5	PF00319; PF01486	II
*HvMADS39*	HORVU.MOREX.r3.5HG0419930.1	5H	1694177–1695760	212	24.61	9.43	55.96	PF00319; PF01486	II
*HvMADS40*	HORVU.MOREX.r3.5HG0494190.1	5H	488377039–488384195	252	29.07	9.05	56.63	PF00319; PF01486	II
*HvMADS41*	HORVU.MOREX.r3.5HG0511210.1	5H	528147816–528157990	330	37.12	9.35	60.49	PF00319; PF01486	II
*HvMADS42*	HORVU.MOREX.r3.5HG0511250.1	5H	528375119–528381095	237	27.34	8.37	54.26	PF00319; PF01486	II
*HvMADS43*	HORVU.MOREX.r3.5HG0523290.1	5H	556038238–556038732	164	18.31	9.89	42.99	PF00319	I
*HvMADS44*	HORVU.MOREX.r3.5HG0523350.1	5H	556216626–556217132	168	18.81	9.09	46.28	PF00319	I
*HvMADS45*	HORVU.MOREX.r3.6HG0540820.1	6H	6878142–6885621	258	28.35	6.29	66.2	PF00319; PF01486	II
*HvMADS46*	HORVU.MOREX.r3.6HG0541730.1	6H	8681080–8681733	217	24.59	9.49	46.97	PF00319	I
*HvMADS47*	HORVU.MOREX.r3.6HG0564200.1	6H	87911081–87913283	244	27.76	6.19	64.63	PF00319; PF01486	II
*HvMADS48*	HORVU.MOREX.r3.6HG0566230.1	6H	100431827–100432921	364	39.05	5.58	38.62	PF00319	I
*HvMADS49*	HORVU.MOREX.r3.6HG0571720.1	6H	135991912–135995133	252	28.37	6.25	62.02	PF00319; PF01486	II
*HvMADS50*	HORVU.MOREX.r3.6HG0604360.1	6H	444223249–444229941	262	29.92	8.9	43.75	PF00319; PF01486	II
*HvMADS51*	HORVU.MOREX.r3.6HG0612320.1	6H	490697619–490706231	241	27.07	9.13	64.86	PF00319; PF01486	II
*HvMADS52*	HORVU.MOREX.r3.6HG0616500.1	6H	512734835–512741542	225	25.18	5.82	48.53	PF00319; PF01486	II
*HvMADS53*	HORVU.MOREX.r3.6HG0620460.1	6H	527947865–527948644	259	27.73	6.06	57.55	PF00319	I
*HvMADS54*	HORVU.MOREX.r3.6HG0624300.1	6H	539666107–539666847	246	27.24	9.02	53.97	PF00319	I
*HvMADS55*	HORVU.MOREX.r3.6HG0624320.1	6H	539783854–539784594	246	27.19	9.1	38.35	PF00319	I
*HvMADS56*	HORVU.MOREX.r3.6HG0624330.1	6H	539816109–539816699	196	21.37	6.07	37.58	PF00319	I
*HvMADS57*	HORVU.MOREX.r3.6HG0624340.1	6H	539865474–539866214	246	27.19	8.92	40.82	PF00319	I
*HvMADS58*	HORVU.MOREX.r3.6HG0624480.1	6H	540393907–540395195	332	36.01	5.99	52.03	PF00319	I
*HvMADS59*	HORVU.MOREX.r3.6HG0624520.1	6H	540477714–540478769	351	38.15	5.81	52.76	PF00319	I
*HvMADS60*	HORVU.MOREX.r3.7HG0651190.1	7H	31957445–31958593	382	42.20	5.8	55.45	PF00319	I
*HvMADS61*	HORVU.MOREX.r3.7HG0651230.1	7H	32091258–32092406	382	42.28	5.56	55.03	PF00319	I
*HvMADS62*	HORVU.MOREX.r3.7HG0653080.1	7H	40028524–40040953	230	25.92	9.07	48.97	PF00319; PF01486	II
*HvMADS63*	HORVU.MOREX.r3.7HG0653160.1	7H	40168716–40186499	230	25.82	8.42	48.28	PF00319; PF01486	II
*HvMADS64*	HORVU.MOREX.r3.7HG0654930.1	7H	44711728–44723317	225	25.95	6.91	57.59	PF00319; PF01486	II
*HvMADS65*	HORVU.MOREX.r3.7HG0658160.1	7H	55100832–55101941	321	35.12	5.16	62.61	PF00319	I
*HvMADS66*	HORVU.MOREX.r3.7HG0658170.1	7H	55198301–55199410	369	40.61	5.36	55	PF00319	I
*HvMADS67*	HORVU.MOREX.r3.7HG0664320.1	7H	80759161–80765868	223	24.93	5.9	53	PF00319; PF01486	II
*HvMADS68*	HORVU.MOREX.r3.7HG0684020.1	7H	205174115–205180772	246	28.55	8.74	52.74	PF00319; PF01486	II
*HvMADS69*	HORVU.MOREX.r3.7HG0684050.1	7H	205774472–205822095	210	23.63	5.53	65.64	PF00319	I
*HvMADS70*	HORVU.MOREX.r3.7HG0705340.1	7H	431301800–431303921	224	25.21	6.53	37.88	PF00319; PF01486	II
*HvMADS71*	HORVU.MOREX.r3.7HG0710700.1	7H	465504510–465505313	267	29.67	10.06	62.46	PF00319	I
*HvMADS72*	HORVU.MOREX.r3.7HG0710730.1	7H	465707479–465708030	183	19.96	8.46	49.7	PF00319	I
*HvMADS73*	HORVU.MOREX.r3.7HG0712380.1	7H	479163044–479164338	360	37.89	5.05	43.49	PF00319	I
*HvMADS74*	HORVU.MOREX.r3.7HG0715350.1	7H	499374226–499375700	420	44.12	5.05	31.88	PF00319	I
*HvMADS75*	HORVU.MOREX.r3.7HG0715360.1	7H	499382256–499383885	443	46.73	4.74	32.87	PF00319	I
*HvMADS76*	HORVU.MOREX.r3.7HG0721170.1	7H	535752621–535758779	232	26.33	9.11	44.5	PF00319; PF01486	II
*HvMADS77*	HORVU.MOREX.r3.7HG0723010.1	7H	545840082–545841893	442	46.93	4.44	28.79	PF00319	I
*HvMADS78*	HORVU.MOREX.r3.7HG0723860.1	7H	548579430–548580392	320	35.51	6.09	47.51	PF00319	I
*HvMADS79*	HORVU.MOREX.r3.7HG0730260.1	7H	577410846–577412070	378	42.20	6.57	63.54	PF00319	I
*HvMADS80*	HORVU.MOREX.r3.7HG0737610.1	7H	597956917–597959338	220	24.71	5.7	62.14	PF00319; PF01486	II
*HvMADS81*	HORVU.MOREX.r3.7HG0737750.1	7H	598228389–598230633	218	24.56	4.74	59.4	PF00319; PF01486	II
*HvMADS82*	HORVU.MOREX.r3.7HG0750110.1	7H	624424643–624425893	416	46.48	5.02	50.28	PF00319	I
*HvMADS83*	HORVU.MOREX.r3.7HG0751440.1	7H	627612677–627613658	185	20.54	9.1	57.2	PF00319	I

### Type I and type II MADS-box genes belong to well-defined subfamilies

**Figure 1:**
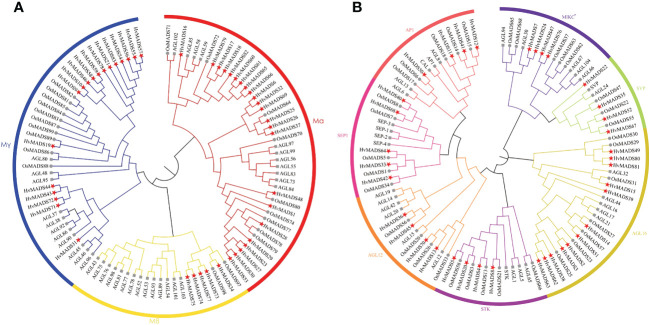
Unrooted phylogenetic tree showing relationships among MADS-box proteins of barley, rice, and Arabidopsis in type I **(A)** and type II **(B)** lineage. The phylogenetic tree was derived with the NJ method in MEGAX. MADS-box proteins from barley are marked with five-pointed star; MADS-box proteins from rice are marked with dot; MADS-box proteins from Arabidopsis are marked with square.

To figure out the phylogenetic relationship of the MADS-box proteins in barley, we separately constructed type I and type II evolutionary trees in terms of the alignment of 83 HvMADS-box genes with rice and Arabidopsis genes (**Table 1**; **Figure 1**). The type I phylogenetic tree showed that the barley genome retained 46 *HvMADS* genes belonging to the Mα, Mβ, and Mγ major subfamilies. The Mα subfamily had the most barley genes and was more closely related to rice genes. The type II phylogenetic tree displayed seven subfamilies, including: AP1, SEPALLATA1 (SEP1), AGAMOUS-LIKE12 (AGL12), SEEDSTICK (STK), AGL16, SHORT VEGETATIVE PHASE (SVP), and MIKC*. In seven subfamilies, barley MADS-box genes were more closely related to rice genes than Arabidopsis. In particular, the AGL16 subclass had the most abundant barley genes and was significantly expanded in barley compared with Arabidopsis and rice (**Figure 1B**).

### Gene cluster, protein motif analysis of HvMADS

**Figure 2:**
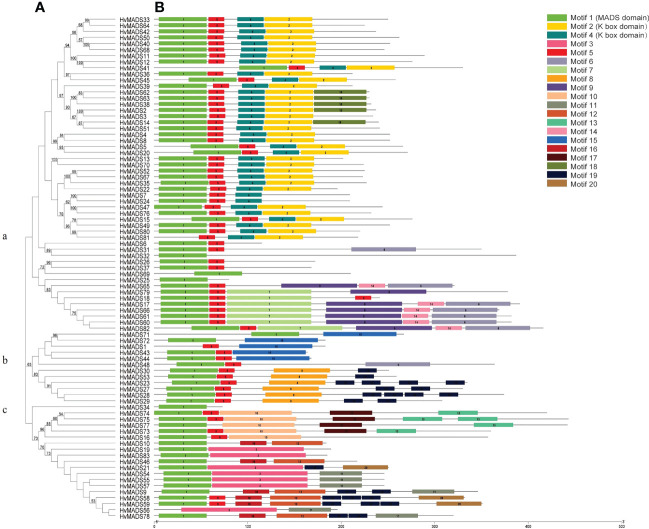
Phylogenetic relationships and motif compositions of HvMADSs. **(A)** The amino acid sequences of the 83 HvMADS proteins were aligned, and the phylogenetic tree was constructed with MEGAX. The tree showed three major phylogenetic subfamilies (a, b, c). **(B)** Schematic structure of the MADS protein motifs identifed in barley. Different motifs were indicated by diferent color boxes.

A phylogenetic tree of HvMADS members was constructed, and this family was divided into three subclasses (**Figure 2A**). The type I *HvMADS* genes were mainly retained in the b and c subclasses, and a small number of them belonged to subclass a, while the type II *HvMADS* genes were mainly retained in subclass a. An online MEME analysis of 83 HvMADS was also conducted, and 20 conserved motifs were determined (**Figure 2B**). The detailed information about the conserved motif, including its width and sequence, is listed in **Table S1**. Each HvMADS contained one to six motifs (**Figure 2B**), and some motifs were common to most members. For example, 96% of HvMADSs contain motif 1, and 80% of HvMADSs contain motif 5. While other motifs were unique to one or several subclasses, such as motifs 2 and 4 (K box domain), they only appeared in subclass a, while motifs 8 and 15 appeared only in subclass b (**Figure 2B**).

### Chromosomal location, gene duplications, and synteny analysis

**Figure 3:**
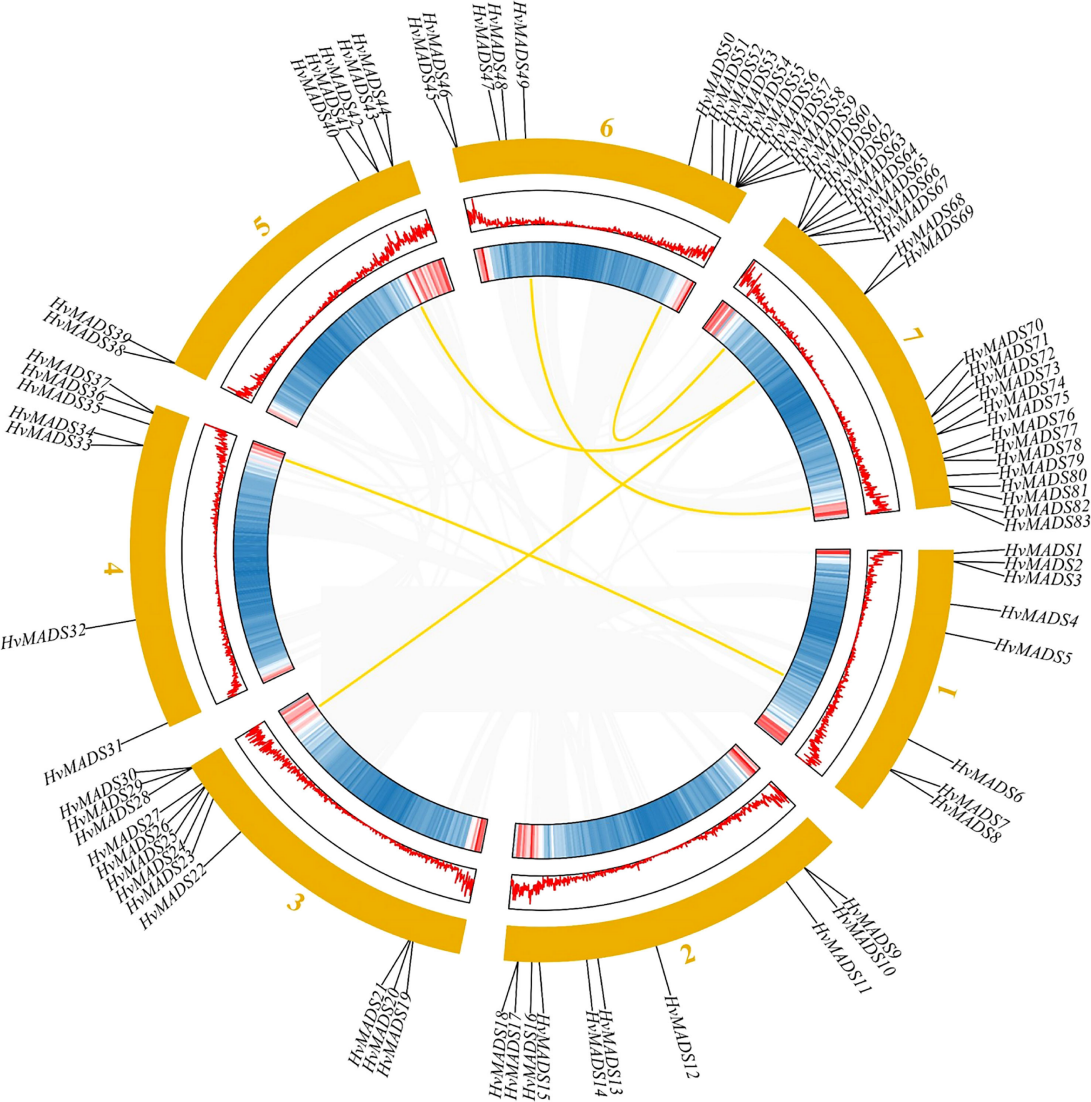
Chromosomal location and gene duplication of *HvMADSs* in the barley genome. The duplicated gene pairs were connected by curved lines.

Eighty-three barley MADS-box genes were generally equally distributed among the seven chromosomes, and genes were named according to their position on the chromosome (**Figure 3**). Interestingly, most genes are located on the distal telomeric ends of chromosomes, and the number of genes on the 1st to the 6th chromosome was distributed almost evenly, except the 7th chromosome contained the most genes compared with other chromosomes (**Figure 3**). On the 7th chromosome, there were 15 type I *HvMADS* genes, which mainly belonged to the Mα subfamily, and nine type II *HvMADS* genes, which mainly belonged to yje AGL16 subfamily (**Figure 3**).

A total of five duplicate gene pairs were identified in *HvMADSs* with BLAST and MCScanX to investigate the expansion of MADS cascade genes in barley (**Figure 3**). Results showed that 26 paralogs composed of 50 *HvMADS* cascade genes were identified. Of these, 21 were tandem duplications, suggesting that tandem repeat duplication was the driving force behind *HvMADS* gene family expansion, and five were segmental duplication events (**Figure 3**). In detail, the five gene pairs were *HvMADS6/HvMADS36*, *HvMADS52/HvMADS67*, *HvMADS40/HvMADS68*, *HvMADS26/HvMADS69*, and *HvMADS49/HvMADS80.* It was noteworthy that on chromosome 7 the largest number of segmental and tandem events occurred, whereas the other tandem duplication blocks were distributed evenly throughout the other chromosomes, of which 2, 2, 2, 2, 3, and 2 paralogous pairs were mapped to chromosomes 1, 2, 3, 4, 5, and 6 respectively (**Figure 3**).

**Figure 4:**
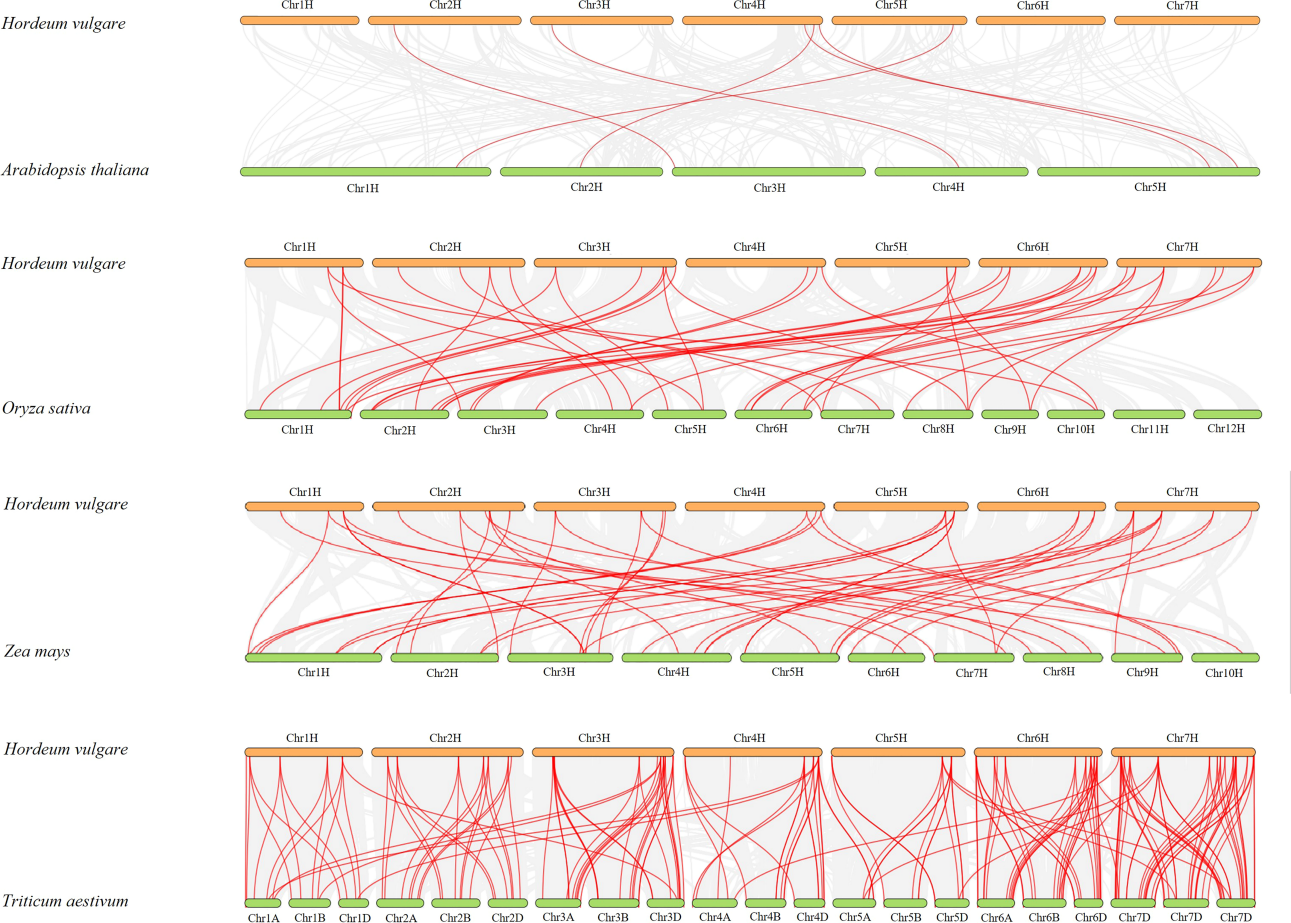
Synteny relationships analysis of *HvMADSs* between *Hordeum vulgare* and *Arabidopsis thaliana, Oryza sativa, Zea mays, Triticum aestivum*.

Syntenic relationships with four other representative species, including *A. thaliana*, *O. sativa*, *Z. mays*, and *T. aestivum*, were compared to determine the mechanisms underlying the evolutionary relationships of *HvMADS* genes (**Figure 4**). Through whole genome-wide syntenic analysis, a total of 6, 43, 46, and 175 orthologous gene pairs between barley and the four compared species were identified as having orthologous counterparts, respectively. In detail, 12 and 11 *HvMADS* genes were orthologous to two copies of MADS genes in rice and maize, respectively. However, there were only two and four *HvMADS* genes orthologous to three copies of MADS genes in rice and maize. On the contrary, comparing barley to wheat, most genes were connected by more than two orthologous gene pairs (**Figure 4**).

### Cis-element analysis of MADS-box family gene promoters in barley

**Figure 5:**
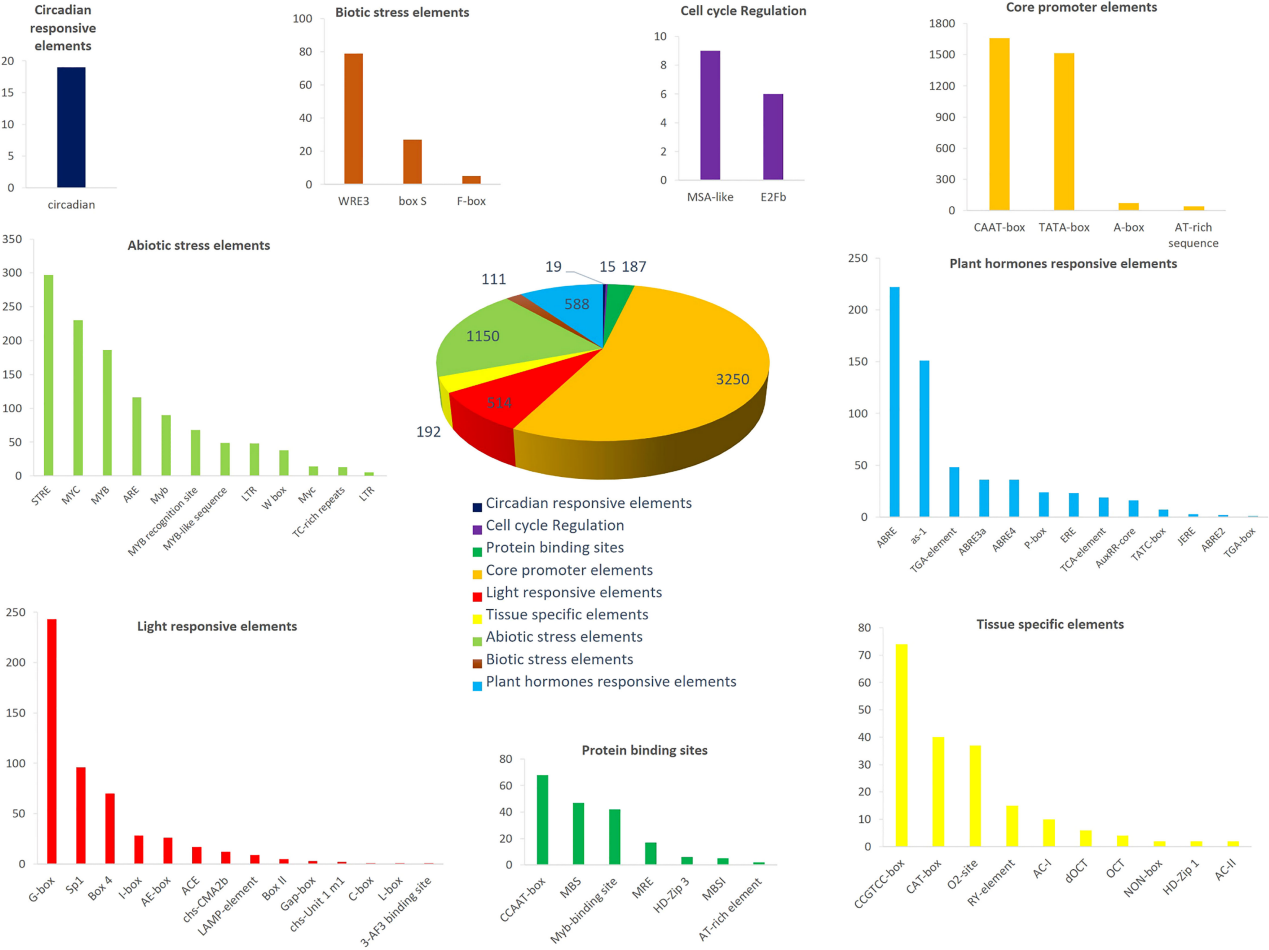
The cis-regulatory elements analysis of *HvMADS* promoter regions. Nine broad categories were predicted including core promoter elements, protein binding sites, hormones responses, tissue specific elements, light responsive elements, abiotic and biotic stress responses, circadian responses and cell cycle regulation elements. The different colors were the various cis-acting elements.

To identify cis-regulatory elements in *HvMADS* genes, we extracted the promoter sequence and analyzed them using the PlantCare server. We categorized all cis-elements into nine broad categories, including core promoter elements, protein binding sites, hormone responses, tissue-specific elements, light-responsive elements, abiotic and biotic stress responses, circadian responses, and cell cycle regulation elements (**Figure 5**; **Table S2**, **Supplementary File**). In the pie chart (**Figure 5**), the proportion of core promoter elements was the greatest, followed by abiotic stress responses, plant hormone-responsive elements, and light-responsive elements. CAAT-box and TATA-box were the most frequently identified core promoter elements (**Figure 5**; **Table S2**). Among the predicted abiotic stress responsive elements, STRE and MYC were the most abundant (**Figure 5**; **Table S2**). Furthermore, we also identified 13 hormone-responsive cis-elements, such as ABRE and as-1, involved in abscisic acid and salicylic acid responsiveness (**Figure 5**).

### Expression analysis of *HvMADS* genes under salt and waterlogging stress

**Figure 6:**
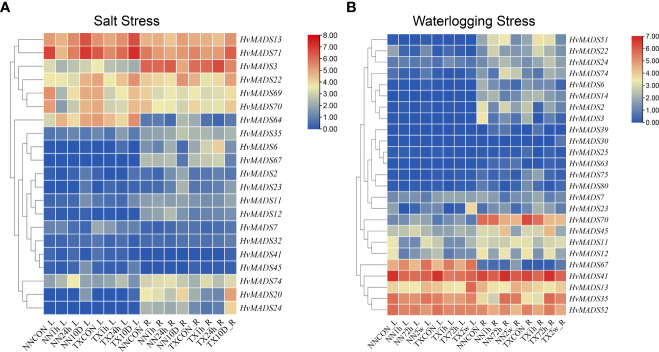
The expression profle of *HvMADSs* in leaf and root of two barley varieties (NN and TX) after 1 h, 24 h, and 10 d of salt **(A)** and 1 h, 72 h, and 2 w of waterlogging **(B)** stress. FPKM values were normalized by log_2_(FPKM) transformation to display the heatmap color scores.

Two barley varieties, NN (Naso Nijo), sensitive to both salt and waterlogging stress, and TX (TX9425), tolerant to salt and waterlogging stress, suffered from 1 h, 24 h, and 10 d of salt stress or 1 h, 72 h, and 2 w of waterlogging stress, respectively. The roots and leaves of each seedling were collected for transcriptome sequencing, and the expressions of 21 and 25 *HvMADS* genes under salt and waterlogging stress, respectively, were analyzed (**Figure 6**). Under salt stress, *HvMADS13* was highly increased after 10 d of treatment in both leaves and roots of two varieties (**Figure 6A**). The expression of *HvMADS70* was repressed by 1 h and 24 h of salt stress but highly induced by 10 d of salt stress in both leaves and roots of two varieties (**Figure 6A**). Strong tissue-specific expression was found in *HvMADS64*, which showed high expression levels responding to salt stress, especially after 10 d of treatment in leaves but this gene was barely expressed in roots in two varieties; however, the expression of *HvMADS70* after waterlogging stress displayed the opposite expression pattern (**Figure 6**). Several *HvMADS* genes were not induced by any abiotic stresses. For example, *HvMADS2*, *6*, and *HvMADS24* displayed almost no expression alteration in response to two treatments in the leaves of two varieties (**Figure 6**). Meanwhile, *HvMADS25*, *30*, *39*, *63*, and *80* were all barely expressed in both leaf and root of two varieties under control or waterlogging stress. In addition, *HvMADS41* was expressed highly in both leaf and root of two varieties under control but depressed by waterlogging stress (**Figure 6B**).

### Protein–protein interaction network for HvMADS under salt and waterlogging stress

**Figure 7:**
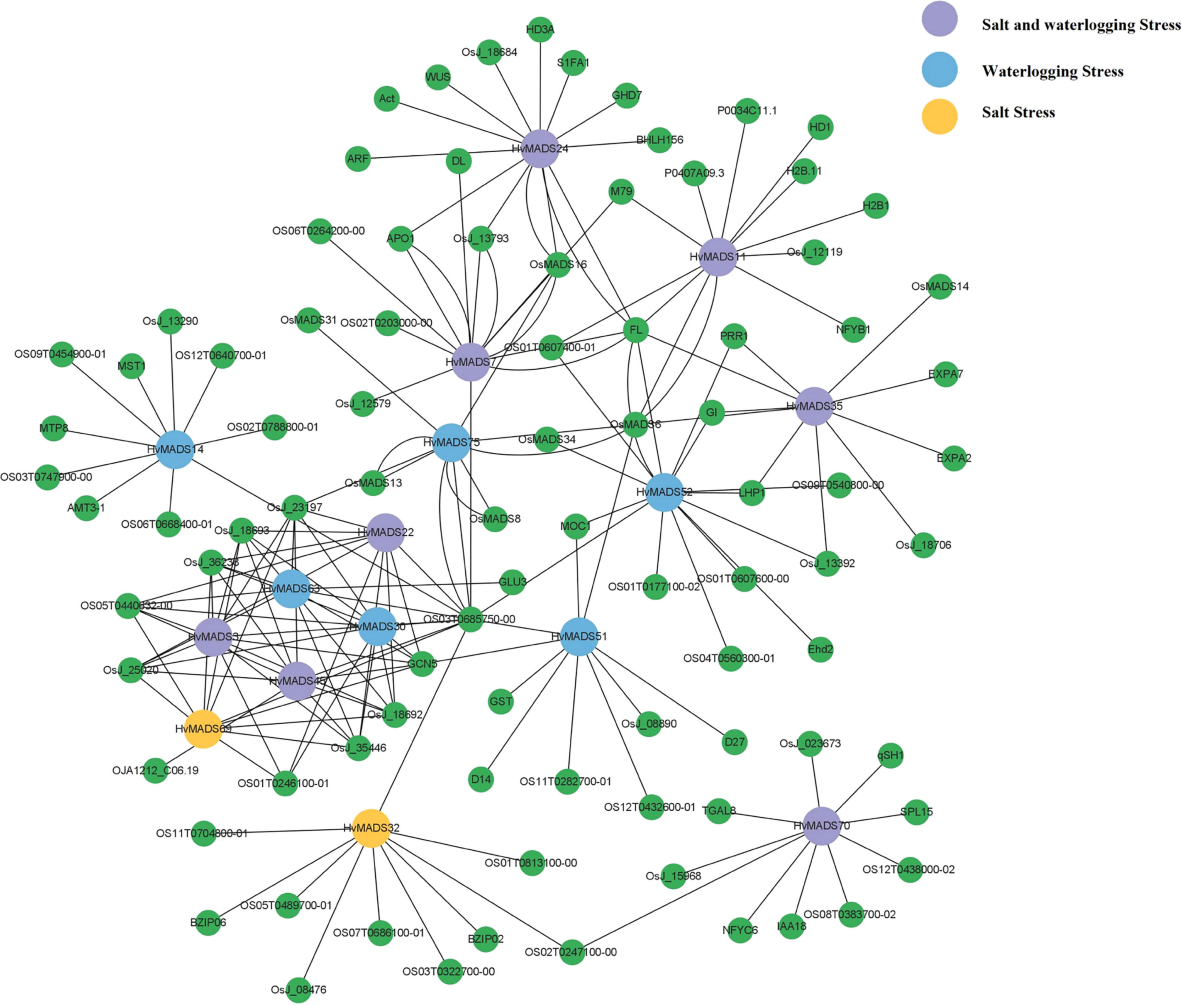
The co-expression regulatory network of MADS cascade genes in barley. Blue dot, HvMADS proteins responsed to waterlogging stress; yellow dot, HvMADS proteins responsed to salt stress; purple dot, HvMADS proteins responsed to both salt and waterlogging stress; green dot, predicted proteins interact with HvMADSs.

We chose the differentially expressed genes as the subject to draw the protein–protein interaction network after salt and waterlogging stress (**Figure 7**). Results found that 10 and 14 *HvMADS* genes homologous to rice and corresponding functional genes with functional interactions were predicted under salt and waterlogging stress, respectively, with eight genes common in both kinds of stress. Interestingly, *HvMADS3*, *22*, *30*, *45*, *63*, *and 69* were closely related to each other, and their interacting genes formed a sub-network. HvMADS11 and 24 were found to interact with NFYB1 (nuclear transcription factor Y subunit B-1) and ARF (auxin response factor), respectively, which were reported to be involved in the salt tolerance mechanism. More HvMADS proteins were predicted and interactions were constructed after waterlogging stress than salt stress in barley (**Figure 7**). In detail, most HvMADS proteins interacted with more than six proteins in the waterlogging stress network, and HvMADS4, 5, and 44 showed the most abundant homologous protein interactions. It was worth noting that HvMADS35 was predicted to interact with EXPA2 and 7 (EXPANSIN), which may cause loosening and extension of plant cell walls for rapid internodal elongation in deep-water rice during submergence. HvMADS35 was also predicted to interact with an myb-like DNA-binding domain-containing protein (OsJ_18706).

## Discussion

### Duplication among type I genes seems to have played major roles in the expansion of MADS-box genes in barley

The annotation of genes has progressed rapidly since the development of plant genome sequencing technology, yet a large percentage of genes remain unclassified. Here, we demonstrated that 83 MADS-box genes in barley were identified, including 46 type I genes and 37 type II genes (**Figure 1**). This number is similar to that of rice, where 75 MADS-box genes were found with 32 type I genes and 43 type II genes (Arora et al., 2007); and to foxtail millet, where 89 MADS-box genes were found with 37 type I genes and 52 type II genes (Lai et al., 2022). Due to the genome’s allohexaploid nature, 300 MADS-box genes were identified in wheat, with 128 type I genes and 172 type II genes (Raza et al., 2022). Kuijer et al. (2021) identified 34 MIKCc MADS-box genes and one pseudogene in barley, while in our study, we identified 33 MIKCc MADS-box genes (**Table 1**; **Figure 1B**). In the former work, 34 MIKCc MADS-box genes were identified by name and BLAST searches, using rice homologues based on *Hordeum vulgare* Morex V1, 2016 and V2, 2019 (Kuijer et al., 2021). In our study, we made a hidden Markov model based on pfam and used this model to search the protein database in the barley genome based on *Hordeum vulgare* Morex V3, 2021. The difference in methods may cause the identification of MIKCc MADS-box genes to be different.

Gene duplication is considered as one of the primary drivers of gene family expansion in plants (Schilling et al., 2018). In this study, we observed the expansion of type I and type II genes in these two lineages (**Figure 1**). There was some disparity in the duplication events between type I and type II genes in barley, rice, and Arabidopsis. For example, duplication events took place with a higher frequency among type I MADS-box genes compared to type II genes in barley and Arabidopsis. Such as *HvMADS54*, *55*, *56*, *and 57* in the Mγ subfamily (**Figure 1A**), which were also in the same class in the HvMADSs cluster (**Figure 2A**), were found as tandem repeat duplications in gene duplication analysis (**Figure 3**). In case of rice, this scenario was reversed, where more type II genes than type I were found in the duplicated segments (**Figure 1**). Gene replication events play pivotal roles in the proliferation of MADS-box genes (Alvarez-Buylla et al., 2000). Our gene duplication analysis showed that 21 of 26 paralogs, including 50 *HvMADS* genes, were tandem duplications (**Figure 3**), suggesting that tandem repeat duplication was the driving force behind the *HvMADS* gene family expansion, which will generate new functionality and enhance the ability of plants to adapt to the environment. Syntenic relationship analysis between barley and Arabidopsis, rice, maize, and wheat (**Figure 4**) showed that *HvMADS* genes had the most syntenic conservation in wheat, and when comparing between barley and wheat, most genes related to more than two orthologous gene pairs, indicating that these genes might be of great significance in MADS family evolution. Altogether, *HvMADS* genes are closer to those in wheat and may evolve from a common ancestor in various plants.

### HvMADSs may interact with plant hormones to defend against abiotic stress

Determining the promoter region features of *HvMADSs* will help us understand the expression patterns of MADS-box genes in barley. A large amount of plant hormone responsive (e.g., abscisic acid, auxin, MeJA, ethylene, and gibberellin) and abiotic stress-responsive (e.g., salt, drought, and hypoxia) cis-elements were found in these promoter regions (**Figure 5**), suggesting that MADS cascade genes are widely involved in regulating the signal transduction network of diverse developmental processes and might have potential functions in stress adaptation and signaling pathways (Zhang et al., 2021). Particularly worth mentioning is that among the stress response elements, 60 *HvMADS* genes, accounting for 72% of the total number of genes, contain ARE response elements (**Figure 5**). ARE response elements are related to anaerobic induction (Dhatterwal et al., 2021), which may imply that most *HvMADS* genes play pivotal roles in waterlogging tolerance networks.

### The possible *HvMADS* genes involved in salt and waterlogging stress

Protein interactions are essential not only for the normal roles that proteins play but also for expanding the functional diversities of proteins (Nobeli et al., 2009). MADS-box genes are widely distributed in a taxonomically broad range of monocot and dicot plant species, and their changes in gene structure, expression, and function have been a major cause of innovations in development during land plant evolution (Theissen et al., 1996; Zahn et al., 2006). MADS-domain transcription factors are key members of regulatory networks underlying multiple developmental pathways and regulatory networks involved in abiotic stress in plants (Ng and Yanofsky, 2001; Hernandez-Hernandez et al., 2007; Callens et al., 2018; Castelan-Munoz et al., 2019). So, it is of great interest and required to exploit fully the potential of MADS-box genes and the protein–protein interaction analysis under abiotic stress for optimizing crop performance. In this study, two barley varieties with contrasting salt and waterlogging tolerance abilities were treated with various treatments. Forty-six HvMADS-box genes were detected in the transcriptome sequencing in response to salt and waterlogging stress, and 16 differentially expressed MADS-box genes were chosen to draw the protein–protein interaction network (**Figures 6**, **7**). So far, Kuang et al. (2019) have found that *HORVU2Hr1G080490.1* (*MADS27*), which was named *HvMADS14* in our study, was upregulated in a salt-sensitive variety but downregulated in a salt-tolerant variety under salt stress in barley. However, in our study, this gene was not detected in the two barley varieties after salt stress (**Figure 6A**). AGL subfamily members are well known for their regulatory roles in salt stress. For example, the expression of *OsMADS26*, the rice AGL12 ortholog, was enhanced by salt stress (Arora et al., 2007). AGL16 has been shown to be a negative regulator, transcriptionally suppressing key components including stress-responsive transcriptional factors and genes involved in ABA signaling and ion homeostasis in salt stress, and may play a role in balancing stress response with growth (Zhao et al., 2021). It was further demonstrated that AGL16 directly binds to the CArG motifs in the promoter of *HKT1;1*, *HsfA6a*, and *MYB102* and repressed their expression (Zhao et al., 2021). In tomato, an AGL15-like gene, *SlMBP11*, was found to code a stress-responsive transcription factor in the positive modulation of salt-stress tolerance, possibly through an abscisic acid-independent signaling network (Guo et al., 2016). In our study, four and 12 *HvMADS* genes were identified in the AGL12 and AGL16 subfamilies, respectively (**Figure 1B**). *HvMADS13* and *70*, which belong to the AGL 12 subfamily, were strongly induced in the leaf of two varieties after 10 d of salt stress (**Figure 6A**); meanwhile, *HvMADS13* also strongly responded to waterlogging stress in the leaf, especially in waterlogging-tolerant variety (**Figure 6B**), suggesting this gene could play key functions in both salt and waterlogging stress. *HvMADS11* was induced by salt stress (**Figure 6A**) and was predicted to interact with NFYB1 (**Figure 7**). NFYB1 was induced under salt stress in soybean, and overexpressing this gene could improve salt tolerance in Arabidopsis (Li et al., 2016). In the SVP subfamily, *HvMADS35* was highly induced in the roots of two varieties after 2 w of waterlogging stress (**Figure 6B**) and was predicted to interact with the myb-like DNA-binding domain-containing protein (OsJ_18706) (**Figure 7**). It was demonstrated that in rice, the gene coding OsJ_18706 protein was significantly downregulated in coleoptiles under submergence and auxin polar transport inhibitors (Wu and Yang, 2020). We also found HvMADS35 worked with EXPA2 and 7, which may cause loosening and extension of plant cell walls for rapid internodal elongation in deep-water rice during submergence (Lasanthi-Kudahettige et al., 2007; Guo et al., 2016). In general, *HvMADS11*, *13*, *and 35* could be candidate genes for further investigation of abiotic stress in barley. Related MADS genes have been found to respond to waterlogging stress in other species. In *Rhododendron hainanense*, nine members of the MADS-box genes showed different degrees of expression after 3 to 20 d of waterlogging treatment (Huo et al., 2021). In rice, *MADS23* was found in response to waterlogging stress (Pandey and Kim, 2012). A member of the MADS box family (MDP0000212925 and AGAMOUS80) was induced under hypoxic conditions in apples (Cukrov et al., 2016). Four genes coding for the MADS-box protein Vrn1 and its homologs were induced under hypoxic treatment in a wheat–sea wheatgrass amphiploid, which showed superior tolerance to waterlogging (Li et al., 2022). In general, MADS-box genes have significantly contributed to abiotic stress in barley, and understanding the MADS-box proteins’ interaction among the diverse networks they are involved in will help to utilize MADS-box genes efficiently in future breeding efforts.

## Conclusion

The MADS-box gene family is not only a central regulator of plant development but is also involved in mediating plant responses or tolerance to a wide range of abiotic stresses as integrators of environmental cues and endogenous hormones in plant species. This study is a comprehensive and systemic analysis of MADS-box genes in barley, where 83 *HvMADS* genes were identified, and phylogenetic relationships and conserved motif analysis all strongly supported the prediction. We also examined their responses to salt and waterlogging stresses, and the stress-responsive genes were identified, which might be exploited for molecular breeding of barley. Finally, the co-expression regulatory network of 16 MADS-box cascade genes was constructed, and we proposed *HvMADS11*, *13*, and *35* as candidate genes for further exploration of their functions under abiotic stress, which will contribute to a better understanding of the MADS-box signal pathways in barley.

## Data availability statement

The data presented in the study are deposited in the NCBI GEO repository, accession number GSE230751.

## Author contributions

Bioinformatics and data analysis were done by FW and ZZ. Study was conceived by YG, LZ, BG, CL, and JZ. Manuscript was written by FW. Supervision and funding by RX. All authors contributed to the article and approved the submitted version.

## Supplementary material

Supplementary Table 1 The MEME motif sequence and width of HvMADSs

Supplementary Table 2 The cis-elements analysis of HvMADS promoters
