# Supplementary material for: Genome-wide analysis of the MADS-box gene family involved in salt and waterlogging tolerance in barley (Hordeum vulgare L.)
Source: Front Plant Sci. 2023 May 9;14:1178065. doi: 10.3389/fpls.2023.1178065 (PMC10203460; doi:10.3389/fpls.2023.1178065)
Supplement: Supplementary Table 1 — The MEME motif sequence and width of HvMADSs [file Table_1.doc]

**Supplementary table 1** The MEME motif sequence and width of HvMADSs

**Supplementary table 2** The cis-elements analysis of *HvMADS* promoters

| Group | Name | Number | Total |
| --- | --- | --- | --- |
| Circadian responsive elements | circadian | 19 | 19 |
| Cell cycle Regulation | MSA-like | 9 | 15 |
|  | E2Fb | 6 |  |
| Protein binding sites | CCAAT-box | 68 | 187 |
|  | MBS | 47 |  |
|  | Myb-binding site | 42 |  |
|  | MRE | 17 |  |
|  | HD-Zip 3 | 6 |  |
|  | MBSI | 5 |  |
|  | AT-rich element | 2 |  |
| Core promoter elements | CAAT-box | 1660 | 3250 |
|  | TATA-box | 1513 |  |
|  | A-box | 74 |  |
|  | AT-rich sequence | 3 |  |
| Light responsive elements | G-box | 243 | 514 |
|  | Sp1 | 96 |  |
|  | Box 4 | 70 |  |
|  | I-box | 28 |  |
|  | AE-box | 26 |  |
|  | ACE | 17 |  |
|  | chs-CMA2b | 12 |  |
|  | LAMP-element | 9 |  |
|  | Box II | 5 |  |
|  | Gap-box | 3 |  |
|  | chs-Unit 1 m1 | 2 |  |
|  | C-box | 1 |  |
|  | L-box | 1 |  |
|  | 3-AF3 binding site | 1 |  |
| Tissue specific elements | CCGTCC-box | 74 | 192 |
|  | CAT-box | 40 |  |
|  | O2-site | 37 |  |
|  | RY-element | 15 |  |
|  | AC-I | 10 |  |
|  | dOCT | 6 |  |
|  | OCT | 4 |  |
|  | NON-box | 2 |  |
|  | HD-Zip 1 | 2 |  |
|  | AC-II | 2 |  |
| Abiotic stress elements | STRE | 297 | 1150 |
|  | MYC | 230 |  |
|  | MYB | 186 |  |
|  | ARE | 116 |  |
|  | Myb | 90 |  |
|  | MYB recognition site | 68 |  |
|  | MYB-like sequence | 49 |  |
|  | LTR | 48 |  |
|  | W box | 38 |  |
|  | Myc | 14 |  |
|  | TC-rich repeats | 13 |  |
|  | LTR | 1 |  |
| Biotic stress elements | WRE3 | 79 | 111 |
|  | box S | 27 |  |
|  | F-box | 5 |  |
| Plant hormones responsive elements | ABRE | 222 | 588 |
|  | as-1 | 151 |  |
|  | TGA-element | 48 |  |
|  | ABRE3a | 36 |  |
|  | ABRE4 | 36 |  |
|  | P-box | 24 |  |
|  | ERE | 23 |  |
|  | TCA-element | 19 |  |
|  | AuxRR-core | 16 |  |
|  | TATC-box | 7 |  |
|  | JERE | 3 |  |
|  | ABRE2 | 2 |  |
|  | TGA-box | 1 |  |
